# Comparative Transcriptome Profiling of mRNA and lncRNA of Ovaries in High and Low Egg Production Performance in Domestic Pigeons (*Columba livia*)

**DOI:** 10.3389/fgene.2021.571325

**Published:** 2021-03-23

**Authors:** Haiguang Mao, Xiuli Xu, Haiyue Cao, Xinyang Dong, Xiaoting Zou, Ningying Xu, Zhaozheng Yin

**Affiliations:** ^1^Animal Science College, Zhejiang University, Hangzhou, Zhejiang, China; ^2^School of Biological and Chemical Engineering, Ningbo Tech University, Ningbo, Zhejiang, China

**Keywords:** lncRNAs, mRNA, egg production, ovary, pigeon

## Abstract

Egg production performance is one of the most important economic traits in pigeon industry. However, little is known regarding how egg production performance is regulated by long non-coding RNAs (lncRNAs) in pigeons. To evaluate the lncRNAs and mRNAs in ovaries associated with egg production performance in domestic pigeons, high-throughput RNA sequencing of ovaries between high and low egg production performance groups were performed and analyzed in this study. A total of 34,346 mRNAs and 24,601 lncRNAs were identified, including 14,525 known lncRNAs and 10,076 novel lncRNAs, of which 811 mRNAs and 148 lncRNAs (*P* < 0.05) were significantly differentially expressed (DE) between the groups of high and low egg production performance. GO and KEGG annotation analysis indicated that the target genes of DE lncRNAs and DE mRNAs were related to cell differentiation, ATP binding and methylation. Moreover, we found that FOXK2, a target gene of lncRNA MSTRG.7894.4, was involved in regulating estrogen receptors. Our study provided a catalog of lncRNAs and mRNAs associated with egg production performance, and they deserve further study to deepen the understanding of biological processes in the ovaries of pigeons.

## Introduction

Egg production performance has been one of the most important economic traits in poultry industry because of the desirable nutritional content of eggs in diets of humans (Du et al., 2020). The continually growing human population leads to an increasing demand for animal products. Therefore, animal production, including egg production, should be increased to meet the consumption needs of the growing human population (Huang et al., 2012). Egg production of poultry mainly depends on the reproductive performance, which is a relatively low heritability trait. Endocrine factors and environmental factors, including illumination time, and feeding allowance could affect egg production (Lewis and Gous, 2006). The egg production performance of egg-laying poultry has progressively been enhanced by traditional breeding and selection methods in the last few decades, but further improvement maximum performance is relatively slow (Zhu et al., 2017). As the female reproductive organ, the ovary has the function of producing and releasing eggs, and acts as an endocrine gland which produces and secretes important reproductive hormones (Zhu et al., 2017). Thus, poultry breeders have focused on the ovaries to study egg production. Moreover, the domestic pigeon is one of the most economically important poultry species in China, as they are a source of meat and eggs, and China is also the largest consumer market for pigeon eggs (Mao et al., 2018). Therefore, it is of great significance to study the regulatory mechanisms of reproduction in ovaries of the pigeon to improve their egg production performance.

Long non-coding RNAs (lncRNAs) are a type of RNA transcripts, which are longer than 200 bases without evident protein-coding capacity (Aliaksandr and Igor, 2015). Accumulating evidences indicate that lncRNAs play important roles in gene expression regulation by directly affecting the process of transcription or recruiting epigenetic complexes (Kung et al., 2013; Mallory and Shkumatava, 2015; Gao et al., 2019). Specifically, the genetic mechanisms of cellular differentiation, cell cycle regulation, epigenetics, and dosage compensation implicate the inhibition of proteins by binding of lncRNAs to miRNAs or to proteins or by titration of miRNAs (Harinarayanan et al., 2018). LncRNAs could also act as guides, signals, scaffolds, and decoys on the basis of their molecular mechanisms (Wang and Chang, 2011). Many researches on lung cancer, liver cancer and other diseases revealed that the expression levels of some specific lncRNAs are significantly correlated with these cancer progressions (He et al., 2019; Pan et al., 2020), rendering them available biomarkers in various types of cancer diagnosis (Maass et al., 2014; Su et al., 2018). LncRNAs have also been found to be key regulators in a wide range of biological processes, including reproduction, but most of the mechanisms of lncRNA related regulation in biological processes remain unclear (Taylor et al., 2015). In the terms of animal genetics, the effects of lncRNAs on economically important phenotypic traits had been studied in the last decade (Zhang et al., 2019; Yang et al., 2020), particularly for the possible application in poultry breeding (Fulton, 2012). However, lncRNAs, as the crucial regulation factors in many biological processes, have not been systematically researched in the ovaries of domestic pigeons related to egg production.

In the present study, we are the first to perform transcriptome analysis of ovaries in pigeons between high and low egg production performance by RNA sequencing. The purpose of this study was to reveal the potential role of the lncRNAs in oogenesis and further provide a new insight in molecular mechanisms involved in the egg production performance in poultries. Our data provide a basis to understand the functional role of lncRNAs in improving egg production performance in pigeons.

## Materials and Methods

### Animal and Ovary Collection

Three female pigeons with the highest and three with the lowest egg laying performance (Table 1), which had the same genetic background, were selected from a pigeonry of 2000 females in Huajia Special Culture Co. (Huzhou, China). All pigeons in this pigeonry were hatched in the same batch and raised one pair (male–female paired) per cage in a windowed house, and fed by a grain-mixed diet consisted of pulses and cereals with 16.89% protein and 11.47 MJ/kg energy content. Reproductive records from the first egg to the second year after birth were recorded in detail. The ovary sample of the selected pigeons was collected and immediately frozen in liquid nitrogen to isolate RNA. All animal procedures were approved by the animal welfare committee of the College of Animal Sciences, Zhejiang University (No. 14814).

**TABLE 1:** Egg production performance in second year after hatching of the six pigeons involved in RNA-seq.

	**High egg production group**			**Low egg production group**		
**Sample**	**HP1**	**HP2**	**HP3**	**LP1**	**LP2**	**LP3**
Egg production	34	40	42	8	12	10
Mean ± SD	38.67 ± 3.40**			10.00 ± 1.63		

### RNA Isolation, Library Preparation, and Sequencing

Total RNA was isolated and purified from each ovarian tissue sample by TRIzol reagent (Invitrogen, Carlsbad, CA, United States) according to the manufacturer’s procedure. The amount and purity of each RNA sample were quantified by NanoDrop ND-1000 (NanoDrop, Wilmington, DE, United States). The integrity of extracted RNA was measured by Agilent 2,100 with RIN number >7.0. About 5 μg of total RNA was used to deplete rRNA following the instruction of the Ribo-Zero^TM^ rRNA Removal Kit (Illumina, San Diego, United States). After depleting rRNAs, the rest RNAs were fragmented into small pieces by divalent cations at high temperatures. Then the cleaved RNA fragments were reversed into cDNA, which was used to synthesize U-labeled second-stranded DNAs with E. coli DNA polymerase I, RNase H and dUTP. The average insert size for the final cDNA library was 300 bp (±50 bp). At last, we performed the paired-end sequencing on an Illumina Hiseq 4000 (LC Bio, China) according to the vendor’s recommended protocol.

### Quality Control and Mapping

Cutadapt was used to remove the low-quality reads, including adaptor contamination, low quality bases, and undetermined bases. FastQC was then used to verify the sequence quality. Bowtie2 (Langmead and Salzberg, 2012) and Hisat2 (Kim et al., 2015) were used to map the reads to the genome of pigeon (*Columba livia*) in NCBI (genome accession number GCF_000337935.1). StringTie was then applied to assemble the mapped reads for each sample. All the transcripts from ovarian tissue samples of pigeons were combined to reconstruct a comprehensive transcriptome by a Perl script. After the final transcriptome was generated, StringTie (Pertea et al., 2015) and Ballgown (Frazee et al., 2015) were used to estimate the expression levels of all transcripts.

### Identification of lncRNAs

Firstly, transcripts shorter than 200 bp and transcripts that overlapped with known mRNAs were discarded. Then CPC (Kong et al., 2007) and CNCI (Sun et al., 2013) were utilized to predict transcripts with coding potential. All transcripts with CPC score =1 and CNCI scores <0 were removed. The remaining transcripts were considered lncRNAs.

### Different Expression Analysis of mRNAs and lncRNAs

StringTie was used to perform expression level for mRNAs and lncRNAs by calculating FPKM. The differentially expressed (DE) mRNAs and lncRNAs were selected with log2 (fold change) >1 or log2 (fold change) =1 and with statistical significance (*p*-value < 0.05) by R package-Ballgown (The significance threshold is coming from an FDR-based adjusted *p*-value).

### Target Gene Prediction and Functional Analysis of lncRNAs

To explore the function of lncRNAs, we predicted the cis-target genes of lncRNAs. lncRNAs might play a cis role acting on the neighboring target genes. In the present study, coding genes in 100 kb upstream and downstream were selected by python script (Wang et al., 2018). Then, we showed functional analysis of the target genes for lncRNAs by the BLAST2GO (Conesa et al., 2005). The Pearson Correlation was calculated through normalized expression values (FPKM), and the correlation *p*-value threshold for selecting lncRNA-mRNA co-expressed pairs was *p*-value < 0.05.

### GO and KEGG Enrichment Analysis

To better understand the biological functions of DE mRNA and lncRNAs of the high and low egg production groups, Gene Ontology (GO) terms and KEGG (Kyoto Encyclopedia of Genes and Genomes) pathway enrichment analysis were performed to explore their biological processes. GO terms were analyzed by using the BLAST2GO. Significance was expressed as a *p*-value < 0.05.

### Validation of RNA-Seq Results by Quantitative Real-Time PCR

For quantitative real-time PCR (qRT-PCR) analysis we randomly selected six lncRNAs (MSTRG.13252.4, MSTRG.13344.3, MSTRG.13479.1, MSTRG.18396.3, MSTRG.20379.2, and MSTRG.9860.1) and 6 mRNAs (CYHR1, HOXC11, TBX18, GM2A, CAMKK2, and NXPH4) that represent differential expression levels of RNA-seq from the ovaries of six individuals. qRT-PCR was performed on an ABI Step One Plus system (Applied BiosystemCarlsbad, CA, United States) by SYBR Premix Ex Taq kit (TaKaRa, Dalian, China) with specific primers (Supplementary Table 1). Gene relative-expression levels were quantified based on β*-actin* gene expression by 2^–Δ^ ^Δ^ ^*Ct*^ method. Three independent biological replicates were used. All the measurements were performed in triplicate.

## Results

### Sequencing Date Summary

Herein, a total of 91.08 Gb raw dates were generated. In detail, 107892662, 94143316, and 99299092 raw reads were obtained for the group of high egg production performance (HP1, HP2, and HP3); 97258276, 101854132 and 106783540 raw reads were obtained for the group low egg production performance (LP1, LP2, and LP3). The raw reads were filtered to obtain clean reads, which were mapped to the Cliv_1.0 version of the pigeon genome sequence, with the mapping ratio ranging from 85.64 to 88.08%. The detailed data are show in Supplementary Table 2. Raw date accession number in GEO is GSE162867, and in SRA is SRP297092.

### Identification of lncRNAs and mRNAs in Pigeon Ovaries

A total of 24,601 putative lncRNAs were identified from the six libraries, including 14,252 known lncRNA and 10,076 novel lncRNAs. In novel lncRNAs, regarding the genomic locations of the lncRNAs, 1,839 were intronic (18.25%), 1,950 were bidirectional (19.35%), 504 were sense (5.00%), 4,919 were intergenic (48.82%), and 864 were antisense lncRNAs (8.57%). Moreover, detailed information on the identified lncRNAs is listed in Supplementary Table 3.

**FIGURE 1:**
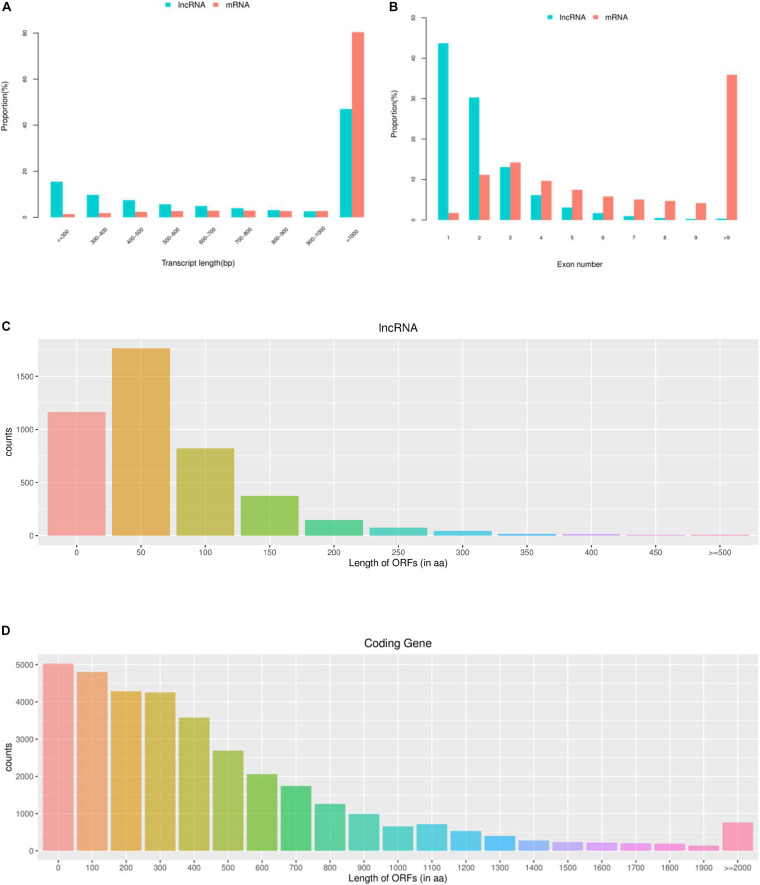
Genomic features of lncRNAs in the ovaries of pigeons. **(A)** The transcript length distribution of lncRNAs and mRNAs. **(B)** The exon number distribution of lncRNAs and mRNAs. **(C)** The ORFs length distribution of lncRNAs. **(D)** The ORFs length distribution of mRNAs.

In the present study, the average length of novel lncRNA transcripts is 1,120 bp, which is shorter than the 4,982 bp length of mRNA transcripts, indicating that lncRNAs in pigeons’ ovaries are shorter than mRNAs (Figure 1A). In addition, the number of exons in lncRNAs was 2.11 on average, which was less than that of mRNAs (9.64 on average). A total of 87.08% lncRNAs have three or fewer exons, while 63.17% of mRNAs have five or more exons (Figure 1B). Moreover, lncRNAs in this study tend to show shorter ORFs than mRNAs in ovaries of piegons (Figures 1C,D).

### Differentially Expressed mRNAs and lncRNAs

**FIGURE 2:**
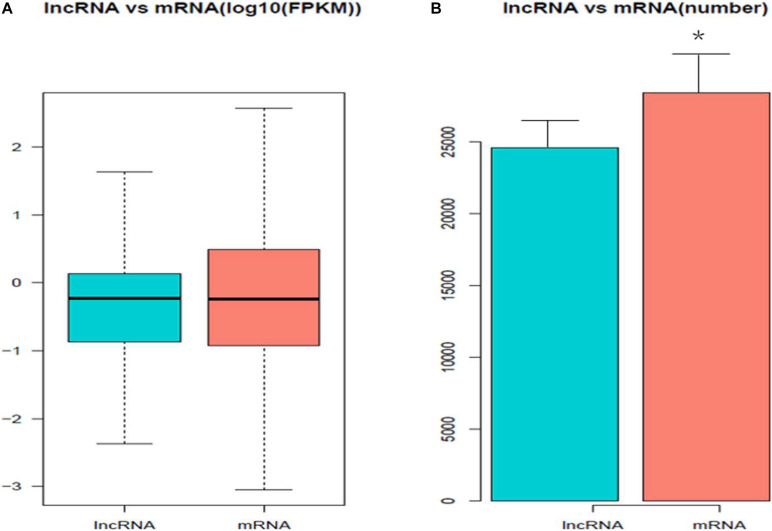
The expression levels and amounts of lncRNAs and mRNAs. **(A)** Boxplots of lncRNAs and mRNAs expression levels (with log10 FPKM method) in the groups of high and low egg production performance. **(B)** The amount of expressed lncRNAs and mRNAs in ovaries with the groups of high and low egg production performance.

To investigate the DE mRNAs and lncRNAs between high and low groups of egg production performance, we examined the DE lncRNAs and DE mRNAs with FPKM levels in ovaries of pigeons. As shown in Figure 2A, the expression levels of mRNAs are higher than those of lncRNAs, and the same trend appears in the number of lncRNAs and mRNAs, the amount of expressed mRNAs is higher than that of lncRNAs (Figure 2B).

**FIGURE 3:**
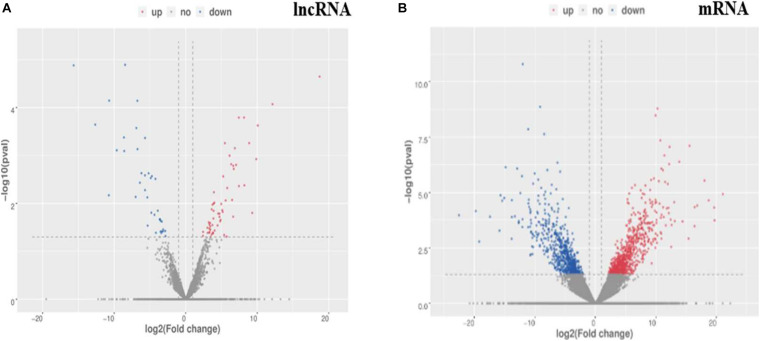
The differential expression of lncRNAs and mRNAs in ovaries between groups of high and low egg production performance. **(A)** Differential expression of lncRNAs. The blue points denote significantly up-regulated lncRNAs, while the red points denote significantly down-regulated lncRNAs in ovaries of high egg production performance. **(B)** Differential expression of mRNAs. The blue points denote significantly up-regulated mRNAs, while the red points denote significantly down-regulated mRNAs in ovaries of high egg production performance.

A total of 811 DE mRNAs (Supplementary Table 9) and DE 148 lncRNAs (Supplementary Table 10) were found between the two groups (the *q*-value is an FDR-based adjusted *q*-value in Supplementary Tables 9, 10). Compared with the low egg production performance group, 90 lncRNAs and 537 mRNAs were significantly upregulated, while 58 lncRNAs and 274 mRNAs were downregulated in the high egg production group. The volcano plot in Figure 3 reveals the DE lncRNAs and mRNAs.

### Functional Enrichment of Differentially Expressed mRNAs

**FIGURE 4:**
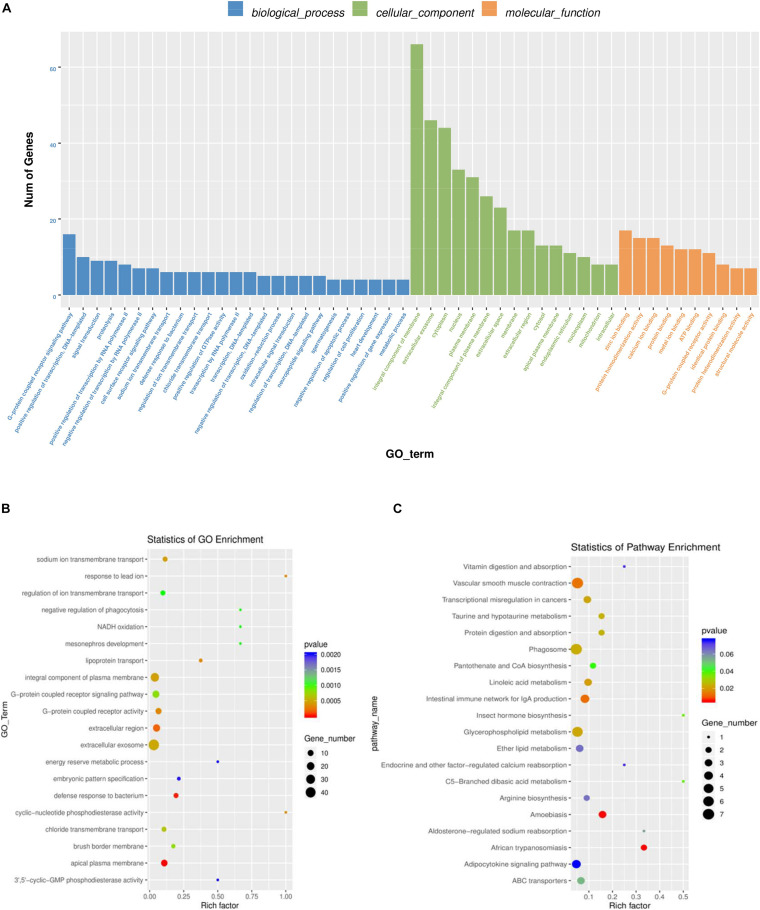
GO and KEGG analysis of differentially mRNAs expression. **(A)** Histogram of GO enrichment of DE mRNAs. **(B)** Scatter plot of GO enrichment for DE mRNAs. **(C)** Scatter plot of KEGG enrichment for DE mRNAs.

Gene Ontology was used to analyze the main functions of DE mRNAs. A total of 1,325 GO terms with functional annotation information are enriched for 811 DE mRNAs. There are 338 GO terms significantly enrich in the GO results that meet the criteria of *P* < 0.05 (Supplementary Table 4). As is shown in Figures 4A,B, the significantly enriched GO terms contain proteolysis, integral component of plasma membrane, zinc ion binding, extracellular exosome, and extracellular region. KEGG pathway analysis reveals 13 significantly enriched pathways (*P* < 0.05), such as linoleic acid metabolism, phagosome, taurine and hypotaurine metabolism and glycerophospholipid metabolism. Detail information is shown in Supplementary Table 5.

### Cis-Regulatory Roles of Differentially Expressed lncRNAs in Ovaries

**FIGURE 5:**
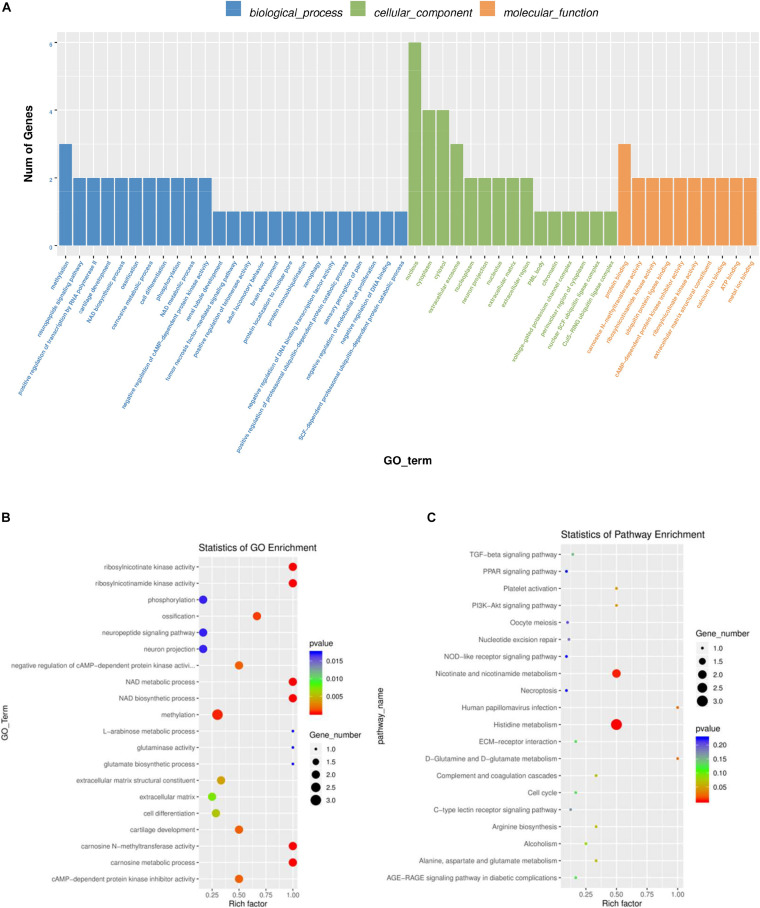
GO and KEGG analysis of differentially lncRNAs expression. **(A)** Histogram of GO enrichment of DE lncRNAs. **(B)** Scatter plot of GO enrichment for DE lncRNAs. **(C)** Scatter plot of KEGG enrichment for DE lncRNAs.

To further explore the regulatory functions of the lncRNAs in the ovaries of domestic pigeons, we predicted the cis-regulated target genes of the DE lncRNAs between the groups of high and low egg production performance. A total of 181 potential lncRNA target genes were revealed in the present study using 100kbp as the cutoff (Supplementary Table 8). Based on these cis-regulated target genes, the results of GO analysis identified 34 significant GO terms (*p* < 0.05) (Supplementary Table 6). The DE lncRNA target genes were revealed to be involved in methylation, phosphorylation, cell differentiation and carnosine metabolic process. The identified cellular component and molecular function categories were mainly related to the nucleus, cytoplasm, cytosol, and protein binging (Figures 5A,B). KEGG pathway enrichment analysis results suggested that the target genes of these lncRNA were mainly involved in histidine metabolism, nicotinate, and nicotinamide metabolism (Figure 5C and Supplementary Table 7).

Based on the predicted results of the DE lncRNA-gene pairs in cis, the first five and the last three lncRNA-gene pairs were listed in Table 2 according to the Pearson Correlation Coefficient. As shown in Table 2, the putative regulation directions of the first five pairs of lncRNA-gene pairs were the same, while those of the last three pairs were opposite.

**TABLE 2:** Differentially expressed lncRNA-gene pairs between high and low egg production groups.

**Gene name**	**lncRNA transcript name**	**Cislocation (bp)**	**Pearson correlation coefficient**
TRIM47	MSTRG.7735.3	1 k	1
FOXK2	MSTRG.7894.4	1 K	1
BACH2	MSTRG.5954.2	1 K	1
LOC110359864	MSTRG.88.13	1 K	1
PPDPF	MSTRG.2346.2	1 K	1
LOC110365815	MSTRG.21861.7	1 K	0.20
DIP2B	MSTRG.15386.5	1 K	−0.29
DIP2B	MSTRG.15386.1	1 K	−0.29

### Co-enriched GO Terms of DE lncRNA and mRNA

A total of ten significantly enriched GO terms in both the DE mRNAs enrichment and the lncRNAs target gene enrichment were identified to reveal the key pathways for regulating egg production performance of domestic pigeons. As shown Table 3, the significantly enriched GO terms contain NAD metabolic process, ribosylnicotinamide kinase activity, methylation, cAMP-dependent protein kinase inhibitor activity and cell differentiation, of which three pathways involve biological process, and the other two pathways involve molecular function.

**TABLE 3:** Co-enriched GO terms of DE lncRNA and mRNA.

**GO term**	**GO function**	***p*-value**
NAD metabolic process	biological_process	0.00
Ribosylnicotinamide kinase activity	molecular_function	0.00
Methylation	biological_process	0.00
cAMP-dependent protein kinase inhibitor activity	molecular_function	0.00
Cell differentiation	biological_process	0.01

### Quantitative Real-Time PCR Validation of Differentially Expressed lncRNAs and mRNAs

**FIGURE 6:**
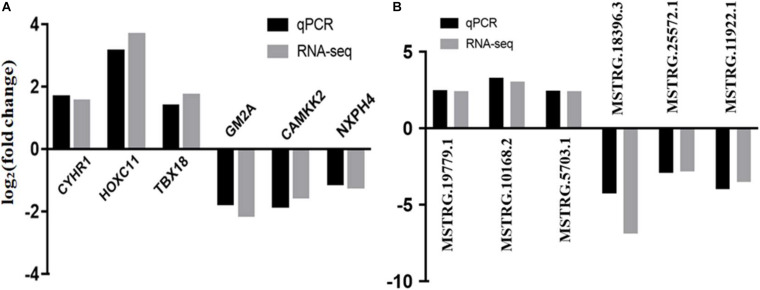
Validation of RNA-seq by qRCR. **(A)** qRCR validation of six mRNAs. **(B)** qRCR validation of six lncRNAs.

**FIGURE 7:**
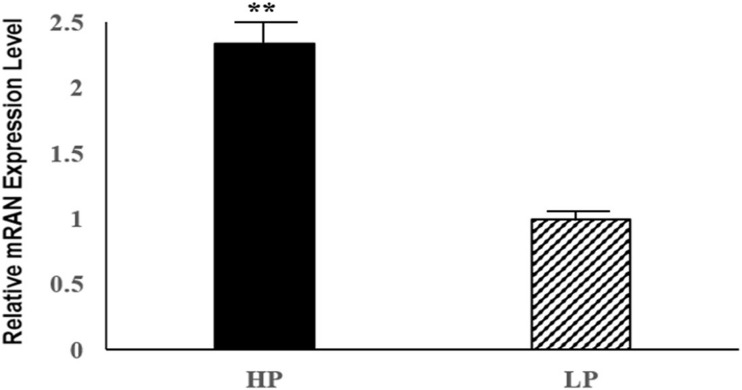
Relative mRNA expression levels of the pigeon ERα gene for HP and LP group. ^∗∗^indicate significant differences (*P* < 0.01).

Six DE mRNAs and six DE lncRNAs were randomly selected to validate the RNA-seq results by qRT-PCR. As shown in Figure 6, the relative fold changes in expression detected by qRT-PCR were consistent with the RNA-seq data, indicating that our transcripts identification and abundance estimation were highly credible. The mRNA expression levels of ERα gene in HP group were significantly higher (*P* < *0.01*) than those in LP group (Figure 7).

## Discussion

Ovary development and egg production are complex biological processes, which are controlled by highly well-coordinated gene regulation, including coding and non-coding RNAs (Ren et al., 2017; Liu et al., 2018). Egg production performance is an important economic trait in the farming of domestic pigeons and a crucial fertility in pigeon production (Yin et al., 2016). To achieve the goal of high egg production, researchers have made great effort on the reproductive regulation mechanisms, which has been driven to screen related genes involved in reproductive regulation of poultries and to mediate the process for improving egg production performance (Liu et al., 2019). In the last few decades, the whole genomes of chickens, ducks, and geese had been published and contributed to facilitate the researches on transcriptome in poultries (Burt, 2005; Huang et al., 2013). Many studies reported that lncRNAs were involved in reproductive regulation, but most of them involved in mammals and plants (Zhang et al., 2014), and whether these lncRNAs worked in the reproductive regulation of domestic pigeons was still unknown. Therefore, in the present study, we performed transcriptome analysis of ovaries in domestic pigeons between high and low egg production performance by RNA sequencing. Specifically, we identified 148 DE lncRNAs and 811 DE mRNAs in ovaries between the groups of high and low egg production performance.

In the last few years, numerous studies had found that lncRNAs played important roles in the oogenesis in ovaries of different species, including pigs, cattle, mice and ducks (Nakagawa et al., 2014; Ren et al., 2017; Yang et al., 2020). The present study is the first to report the transcriptome profiling of lncRNAs and mRNA in the ovaries of pigeons. The results revealed that the lncRNAs identified in our study showed fewer exons and shorter lengths of transcripts, which were consistent with other species reported in the previous studies (Shen et al., 2016), indicating that the lncRNAs sequencing results obtained in our study were reliable. The RNA-seq results showed that 52.96% of lncRNAs were shorter than 1000 bp, while 80.42% of mRNAs were longer than 1000 bp. What’s more, the average expression levels of lncRNAs were higher than those of mRNAs in pigeon ovaries, indicating that the lncRNAs in pigeon ovaries might play important roles in egg production.

Studies had confirmed that various signaling pathways and regulatory mechanisms were participated in the regulation of egg production (Ren et al., 2017; Tao et al., 2017). In this study, we applied GO terms and KEGG pathways analysis to further reveal the biological functions of the target genes of DE lncRNAs and DE mRNAs related to egg production in pigeon ovaries. The results showed that both of these lncRNAs and mRNAs were involved in the regulation of ATP binding, protein binding, cell differentiation and transcription by RNA polymerase II. For example, ATP binding has been shown to be involved in the regulation of oocyte formation (Quiroz et al., 2020). The RNA polymerase II involved in a combinatorial control of the timely regulated Spo11 splicing during the process of meiosis (Cesari et al., 2020).

Most evidence suggested that the expression of lncRNAs could regulate and had high correlations with expression of the neighboring mRNAs by transcriptional coactivation or repression (Ponting et al., 2009; Engreitz et al., 2016). Thus, we supposed that there was a mechanism that the lncRNAs could affect the oogenesis and egg production by mediating the putative regulation of corresponding target mRNAs in pigeon ovaries. In the present study, the DE cis-target genes, located within 100 kb upstream and downstream of the 148 DE lncRNAs, were used to predict the potential functions in the putative regulation of egg production in pigeons. The result revealed that the DE coding gene forkhead box K2 (FOXK2) might be regulated by the DE lncRNA MSTRG.7894.4, and FOXK2 was down-regulated in HP ovaries.

Forkhead box K2, also known as ILF or ILF1, is an important member of the forkhead transcription factors, containing a conserved forkhead winged helix-turn-helix DNA binding domain (FOX domain) (Zhang et al., 2018). The forkhead transcription factors are considered as an evolutionarily conserved family of proteins. In common with other forkhead transcription factors, FOXK2 also contains a FOX domain in addition to a FHA domain, which regulates its interactions with other proteins (Marais et al., 2010). In the last few decades, over 40 different forkhead transcription factors had been indentified in mammals, and these proteins played important roles in numerous cellular processes, including development, growth, reproduction, cell proliferation and cycle by regulating the expression of their respective target genes (Myatt and Lam, 2007; Hannenhalli and Kaestner, 2009). Previous studies had shown that the FOXK2 interacted with estrogen receptor α (ERα), and inhibited the ERα-regulated transcriptional activity through enhancing the ubiquitin-mediated protein degradation of ERα (Liu et al., 2015). The above process involved the interactions between FOXK2 and BRCA1/BARD1, which was the E3 ubiquitin ligase of ERα (Baer and Ludwiq, 2002). FOXK2 interacted with BARD1 and served as the scaffold protein for BRCA1/BARD1 and ERα, resulting in the enhancing degradation of ERα, which ultimately accounted for the decline of its transcriptional activity (Liu et al., 2015). It is well known that estrogen, as an important female reproductive hormone, plays a key role in the reproductive process of female animals by binding to its receptor, estrogen receptor α, so as the egg production process of poultries (Guo et al., 2020). In addition, ERα could control gonadotropin-regulated oocyte maturation by regulating the expression of NPPC and NPR2 (Liu et al., 2017). Our result showed that the mRNA expression levels of ERα gene in HP group were significantly higher (*P* < 0.01) than those in LP group, thus we suspected that FOXK2 might influence the effect of estrogen and the process of oocyte maturation in pigeon ovaries by regulating the transcriptional activity of ERα. Therefore, we inferred that the FOXK2 gene could be a candidate gene for further study in terms of how it affects egg production performance.

In conclusion, this study is the first comprehensive description of mRNA and lncRNA profiles of ovaries from pigeons with high and low egg production performance. A number of DE mRNAs and lncRNAs are revealed to be associated with egg production performance in domestic pigeons. Moreover, the DE lncRNAs found in this study could provide new insights of further understanding the mechanism of egg production in ovaries in poultry. The lncRNA MSTRG.7894.4 might play an important regulatory role in egg production by affecting its potential target gene FOKX2. Therefore, lncRNA MSTRG.7894.4 might be a potential candidate lncRNA for regulating egg production in pigeons, and more detailed studies are required.

## Data Availability Statement

The data presented in the study are deposited in GEO and SRA repository, the accession number in GEO is GSE162867, and in SRA is SRP297092.

## Ethics Statement

The animal study was reviewed and approved by the animal welfare committee of the College of Animal Sciences, Zhejiang University.

## Author Contributions

HM analyzed the data as well as he drafted the manuscript. XX performed the RNA extraction and qRT-PCR. HC collected the tissue samples. XD and XZ provided suggestions for this study. NX and ZY conceived the project and designed the experiments. All authors contributed to the article and approved the submitted version.

## Conflict of Interest

The authors declare that the research was conducted in the absence of any commercial or financial relationships that could be construed as a potential conflict of interest.

## Supplementary Material

Supplementary Table 1 Primers of lncRNAs and mRNAs used in qPCR.

Supplementary Table 2 Quality control of the sequencing data.

Supplementary Table 3 The information for the identified lncRNAs.

Supplementary Table 4 GO terms analysis of differentially mRNAs expression.

Supplementary Table 5 KEGG pathways analysis of differentially mRNAs expression.

Supplementary Table 6 GO terms analysis of differentially lncRNAs expression.

Supplementary Table 7 KEGG pathways analysis of differentially lncRNAs expression.

Supplementary Table 8 LncRNAs-mRNAs significantly correlated pairs.

Supplementary Table 9 Differentially expressed genes.

Supplementary Table 10 Differentially expressed lncRNAs.
